# Posterior Reversible Encephalopathy Syndrome in a Patient with Hemorrhagic Fever with Renal Syndrome

**DOI:** 10.1155/2020/1017689

**Published:** 2020-02-28

**Authors:** Ermira Muco, Amela Hasa, Arben Rroji, Arta Kushi, Edmond Puca, Dhimiter Kraja

**Affiliations:** ^1^Department of Infectious Diseases, Hospital University Center “Mother Theresa”, Tirana, Albania; ^2^Department of Imaging Sciences, Hospital University Center “Mother Theresa”, Tirana, Albania

## Abstract

We presently report the case of hantavirus infection in a 45-year-old male who was hospitalized to our clinic of infectious diseases, with fever, myalgia, vomiting, nausea, headache, and abdominal pain. The physical findings included hepatomegaly, splenomegaly, rash, and conjunctival injection. Eight days before the start of complaints, the patient has cut trees in the mountain. An acute renal failure was observed with an oliguria and an increase of serum creatinine and blood urea nitrogen. Urinalysis shows albuminuria and hematuria. Elevations of amylase, lipase, and liver enzymes levels, low serum albumin level, and thrombocytopenia were observed. A positive ELISA test for hantavirus IgM/IgG antibodies confirmed hemorrhagic fever with renal syndrome. On the third day of hospitalization, the patient had seizures. The unenhanced head computed tomography (CT) performed after seizures showed subcortical bilateral hypodensities within frontal, parietal, and occipital regions corresponding to areas of increased signal intensity in magnetic resonance imaging (MRI) associated with cerebral edema in posterior reversible encephalopathy syndrome (PRES). The treatment consisted of supportive therapy. The patient underwent another head MRI with contrast enhancement after 2 months, which resulted normal.

## 1. Introduction

Hantaviruses are enveloped RNA viruses and members of the Bunyaviridae family. Hantavirus infection to humans is considered a spill over infection that causes two types of serious illnesses, hemorrhagic fever with renal syndrome (HFRS) and hantavirus pulmonary syndrome (HPS) [1]. People can also become infected when they touch mouse or rat urine, droppings, or nesting materials that contain the virus and then touch their eyes, nose, or mouth. Hantavirus infection affects 30,000 individuals annually and tends to occur among people living in lower socioeconomic housing environments and those enjoying the outdoors [2]. The species that cause HFRS include Hantaan River, Dobrava-Belgrade, Saaremaa, Seoul, Puumala, and other hantaviruses. These are found in Europe, Asia, and Africa [3]. Hantaan and Dobrava virus infections usually cause severe symptoms, while Seoul, Saaremaa, and Puumala virus infections are usually more moderate [4]. Cases with hemorrhagic fever with renal syndrome in Albania are caused by Dobrava strains [5]. Albania, a part of Balkans, is part of an endemic area [6]. Posterior reversible encephalopathy syndrome (PRES) was first described in 1996 and is a clinico-radiological syndrome characterized by symptoms including a headache, seizures, altered consciousness, and visual disturbances [7]. Infections are one of the clinical conditions associated with PRES.

## 2. Case Report

**Figure 1:**
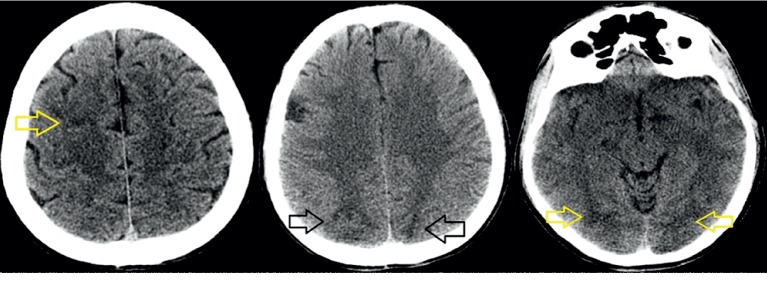
CT scan images: bilateral subcortical hypodensities in frontal, occipital, and parietal regions.

**Figure 2:**
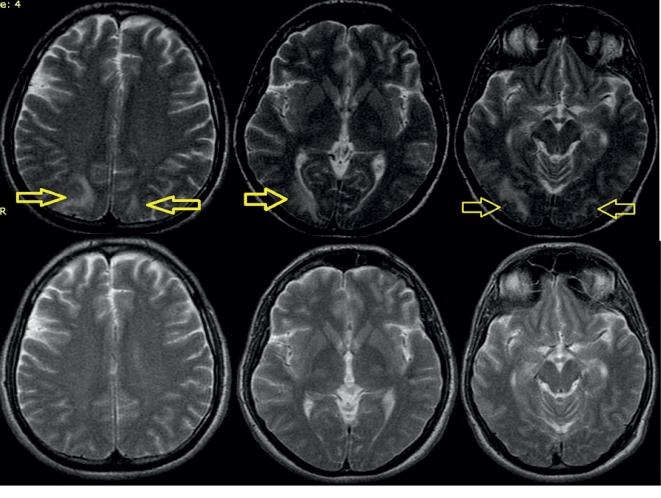
Axial T2 images: bilateral hyperintense zones in parietal and occipital regions. Comparative pictures (lower) showing total disappearance of lesions after 2 months.

**Figure 3:**
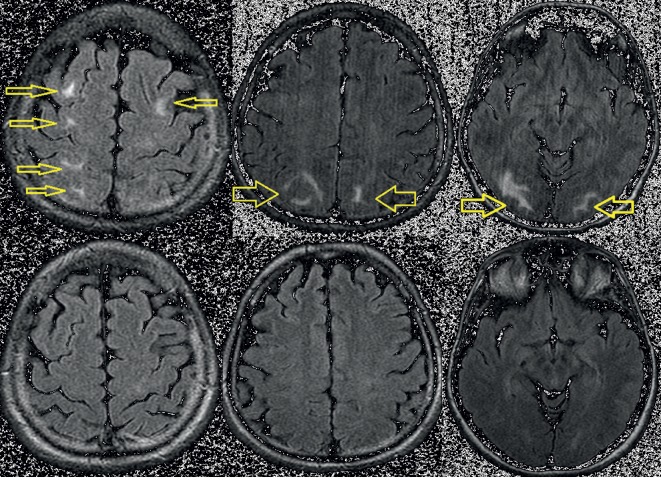
Axial FLAIR: bilateral hyperintense zones in frontal, occipital, and parietal regions. Comparative pictures (lower) showing total disappearance of lesions after 2 months (resolving vasogenic edema).

Our case is a 45-year-old white male who was hospitalized to the clinic of infectious diseases, with fever (39°C), myalgia, vomiting, nausea, headache, and abdominal pain. The physical findings included hepatomegaly (19 cm), splenomegaly (16 cm), rash, and conjunctival injection. Eight days before the start of complaints, the patient had been cutting trees in the forest. He did not have a history of traveling to another HFRS endemic area. An acute renal failure was observed in the laboratory tests with an increase of serum creatinine and blood urea nitrogen. Urinalysis shows albuminuria (9.9 gr) and hematuria (35–40 cell/field). Initial total blood count revealed thrombocytopenia (91,000/mm^3^). Elevations of amylase, lipase, aspartate aminotransferase (AST), and alanine aminotransferase (ALT) levels and low serum albumin level were observed as shown in Table 1. PCR was 11.4 mg/L. Also, an oliguria (300 ml/day) was present. On the third day of hospitalization, the patient had seizures. He was transferred to the Intensive Care Unit because of his worsening condition. The patient refused to have a lumbar puncture. The unenhanced head CT performed in urgency conditions after seizures showed subcortical bilateral hypodensities within frontal, parietal, and occipital regions (Figure 1). A head MRI with intravenous contrast showed hyperintensities in affected regions in T2 and FLAIR sequences without diffusion restriction of signal and without microhemorrhages in T2*∗* sequences (Figures 2 and 3). The radiological consultations considered these pathological images as edematous regions which correspond with posterior reversible encephalopathy syndrome. The electroencephalogram realized found problems related to electrical activity of the brain: “Intermittent bilateral 7-8 Hz slow wave on the left temporal and frontal lobe in a background of low amplitude registration.” HFRS was detected from a blood sample drawn two days after hospitalization, with a positive ELISA test for hantavirus IgM and IgG antibodies. First blood sample showed hantavirus IgM antibody titer 8.2 (0.9–1.1) and IgG antibody titer 6.7 (0.9–1.1). Second blood sample evaluation, after two weeks, showed hantavirus IgM antibody titer 7.1 and IgG antibody titer 6.9. Serological test of Leptospira, HBV (anti-HBc antibody test and HbsAg antigen test), and HCV (anti-HCV antibody test) resulted negative. All the laboratory test results during hospitalization are shown in Table 1. Treatment consisted of supportive therapy with ceftriaxone, corticosteroids, antiepileptic, saline infusions, electrolytes, antipyretics, and oxygen therapy. The patient was discharged after 16 days. He underwent another head MRI after 2 months, which resulted normal, without presence of any cerebral hyperintensities (Figures 2 and 3).

## 3. Discussion

Hantaviruses have a worldwide distribution and are broadly split into the New World hantaviruses, which includes those causing HPS, and the Old World hantaviruses (including the prototype Hantaan virus (HTNV)), which are associated with a different disease, hemorrhagic fever with renal syndrome (HFRS) [8]. Epidemic seasonal predominance was observed in autumn/winter [9]. Our case was introduced in summer. Summer as the season of occurrence of the disease is also described in other articles [10]. Forestry workers and farmers have an increased risk of exposure. Even our patient worked in the forest cutting trees. Incubation of HFRS infection has not been precisely determined, but it is most frequently around two weeks. Patients with HCPS typically present a short febrile prodrome of 3–5 days [11]. In addition to fever and myalgias, early symptoms include headache, chills, dizziness, nonproductive cough, nausea, vomiting, and other gastrointestinal symptoms. Malaise, diarrhea, and lightheadedness are reported by approximately half of all patients, with less frequent reports of arthralgia, back pain, and abdominal pain [1]. Conjunctival, cerebral, and gastrointestinal (GI) hemorrhages occur in about one-third of patients [4]. The basic pathologic and pathophysiologic disorder in HFRS is capillary damage (vasculitis) [12]. Increased vascular permeability and decreased platelet count are the hallmarks of hantavirus-associated diseases [1]. The diagnosis of hantavirus infections in humans is based on clinical and epidemiological information, as well as laboratory tests. We review diagnosis for hantavirus infections based on serology (ELISA IgM and IgG tests were used for the detection of specific IgM and IgG antibodies), PCR, immunochemistry, and virus culture [13]. We could not perform the hantaan virus PCR test in Albania. Posterior reversible encephalopathy syndrome (PRES) is a neurotoxic state with a mechanism not well understood but is thought to be related to the altered integrity of the blood brain barrier. A hallmark of pathogenesis is increased vascular permeability that seems to be due to endothelial cell dysfunction [14]. In PRES, most commonly, there is vasogenic edema within the occipital and parietal regions (∼95% of cases), usually symmetrical. PRES can be found even in a nonposterior distribution, mainly in watershed areas, including within the frontal, inferior temporal, cerebellar, and brainstem regions. PRES presents with rapid onset of symptoms including headache, seizures, altered consciousness, and visual disturbances [15–17]. In our case, the patient presented with seizures after three days of hospitalization. Infection may be an important cause of PRES. Treatment of hantavirus infections is mainly supportive and involves intensive medical care. Our case discharged from hospital in a good condition. MRI of head realized after 2 months resulted normal. If promptly recognized and treated, the clinical syndrome usually resolves within a week and the changes seen in MRI resolve over days to weeks.

## 4. Conclusion

In summary, hantavirus infection should be considered in the differential diagnosis of renal failure, especially in patients from endemic areas and typical history. The diagnosis is established with laboratory techniques. In cases of neurological symptoms, realization of CT scan and MRI head is useful to detect PRES. Treatment is mainly supportive and involves intensive medical care.

## Figures and Tables

**Table 1:** Laboratory data of biochemical and clinical tests.

Laboratory data	Reference range	D0	D1	D2	D3	D5	D7	D14
AST	0–35 U/L	154	113	162	87	77	77	69
ALT	0–45 U/L	97	90	133	97	87	127	150
Bilirubin	<1.2 mg/dL	0.3	0.5	0.6	0.4	0.6	0.5	
Alkaline phosphatase	32–117 U/L	46	47	46	42	66	66	77
Amylase	28–100 U/L	—	—	153	110	—	—	195
Lipase	21–67 U/L	—	—	224	146	—	—	259
Gamma GT	0–55 U/L	72	109	102	98	122	137	199
Lactate dehydrogenase	125–250 U/L	435	334	438	597	338	255	212
Albumin	3.5–5.2 g/dL	2.8	2.8	2.8	2.4	3.1	3.1	3.6
Total protein	6–8.3 g/dL	5.3	5.2	5.3	4.9	6	6.1	6.8
Serum creatinine	0.1–1.3 mg/dL	6.9	7.5	7.6	4.8	3.2	1.7	0.9
Blood urea nitrogen	<43 mg/dL	193	187	236	169	104	67	36
Creatinine kinase	0–171 U/L	53	75	219	642	1902	435	75
Glucose level	74–106 mg/dL	196	163	142	147	162	145	98
Platelet count	150–390 × 10^3^/mm^3^	91	94	133	212	294	261	178
White blood cells	4000–10,000/mm^3^	11.2	9.1	9.2	9.8	12.6	9.7	10
Hematocrit	35–50%	40	41.7	35.9	40	41.2	41.8	43.7
